# Social media, adolescent mental health, and diet: a policy content analysis

**DOI:** 10.1186/s12889-026-26786-7

**Published:** 2026-03-12

**Authors:** Kaitlin Conway-Moore, Laurence Blanchard, Anaely Aguiar, Jennifer O’Mara, Ioana Vlad, Harry Rutter, Cécile Knai

**Affiliations:** 1 Faculty of Public Health Policy, London School of Hygiene and Tropical Medicine, 15-17 Tavistock Place, London, WC1H 9SH UK; 2 System Dynamics Group, Department of Geography, University of Bergen, Bergen, Norway; 3 World Cancer Research Fund International, London, UK; 4 Department of Social & Policy Sciences, University of Bath, Bath, UK

**Keywords:** Adolescents, Social media, Mental health, Diet, Policy content, Document analysis

## Abstract

**Background:**

Alongside the benefits of social media for information sharing and social connection, recent evidence suggests it can also have harmful impacts on adolescent mental health and diet, including anxiety, depressive and disordered eating symptoms, and body dissatisfaction.

**Methods:**

Using document analysis, this study examines how recent strategies, action plans, policies and guidelines released by international organisations leading in the areas of child and adolescent health capture and address the links between adolescents’ social media use and both their mental health and diet outcomes. Summative content analysis was used to catalogue the inclusion of terms related to social media, adolescent mental health and adolescent diet in each document, and individual policy recommendations and actions were then extracted and categorised to identify themes relevant to current adolescent health priorities that can be leveraged to address the negative impacts of social media use among this age group.

**Results:**

Of the 31 policy documents analysed across 11 organisations, only one included explicit mention of a negative association between social media and both adolescent mental health and diet, while none made recommendations to address it. Emerging themes related to priorities for adolescent health included health services provision, knowledge promotion, online content regulation and environmental improvements, all of which have the potential to serve as leverage points to act on recent evidence related to adolescent social media use.

**Conclusions:**

While strategies to address the harmful effects of adolescent social media use on both mental health and diet are largely missing among the recent outputs of leading international organisations, their existing priorities for adolescent health, combined with emerging online tools and national legislation, may provide helpful paths forward.

**Supplementary Information:**

The online version contains supplementary material available at 10.1186/s12889-026-26786-7.

## Background

Over the last decade, the amount of time people spend online has dramatically increased. This is particularly true for adolescents, with social media use now an essential part of many of their lives. In 2023, a United States Surgeon General’s Advisory found that 95% of 13–17-year-olds use social media, with more than a third reporting near constant use. This report also found that 40% of 8–12-year-olds use social media, despite the minimum age for creating an account on most platforms being 13 years [1]. Although social media has many benefits for young people, including fostering connections across geographic boundaries, building social support, and providing access to information, recent evidence suggests it also has the potential to be harmful to both their mental and physical health [2, 3].

Confronting obesity: Co-creating policy with youth (CO-CREATE) was a European Commission-funded research project that ran from 2018–2023 and engaged young people to identify the factors driving adolescent obesity and devise potential policy-driven solutions to address it [4]. In systems mapping exercises with 16–18-year-olds in five European and one African country as part of CO-CREATE, participants consistently identified social media—and in particular exposure to influencers and celebrities on social media—as having a negative impact on their mental health, including poor body image, self-esteem, stress, interpersonal relationships and loneliness, anxiety and depressive symptoms [5, 6]. This impact on mental health was in turn identified by these young people as an impetus for excessive and compulsive dietary intakes and reduced motivation to exercise or consume a healthy diet.

Youth perspectives related to the harmful impacts of social media for mental health and diet captured in the CO-CREATE systems mapping work were further supported by a recent systematic review by CO-CREATE researchers Blanchard et al., which included 21 studies from across 12 countries and participants ranging in age from 10–19 years [7]. In this review, which looked at a broad range of mental health and diet outcomes, it was found that social media use was positively associated with anxiety (3 studies, *n* = 1,141 participants), depressive symptoms (five studies, *n* = 308,812 participants), disordered eating symptoms (i.e., compulsive overeating: four studies, n = 9,068 participants; weight loss/control: four studies, *n* = 59,047 participants; and general disordered eating: 6 studies, *n* = 4399 participants), and body dissatisfaction (4 studies, *n* = 5469 participants). Along with this, four of the included studies (*n* = 6,185 participants) indicated pathways of effect between social media and dietary outcomes such as binge eating, emotional eating, respect for hunger and fullness cues and dietary restraint, with mental health indicators such as anxiety, body image and self-esteem acting as a mediator in these relationships. In order to examine social media’s impact on adolescent health inequalities, the study also assessed mental health and diet outcomes according to PROGRESS-Plus health equity characteristics [8]. It was found that only the role of sex/gender was measured across the included studies (*n* = 8 out 21), and results were inconclusive.

It is important to note that most of the existing evidence presented above relates to correlational rather than causal findings. However, while pointing to the need for more research designed to establish causality in the relationship between adolescent social media use and negative mental health and diet outcomes, this growing body of international evidence also indicates an emerging public health issue that requires attention from relevant bodies interested in adolescent health and wellbeing. As per the 2023 US Surgeon’s Advisory: “At this time, we do not yet have enough evidence to determine if social media is sufficiently safe for children and adolescents. We must acknowledge the growing body of research about potential harms, increase our collective understanding of the risks associated with social media use, and urgently take action to create safe and healthy digital environments […]” [1]. To this end, the present study aims to further build on the work of the CO-CREATE project to explore the extent to which recent strategies, action plans, policies and guidelines put forth by international organisations with significant influence in the areas of child and adolescent health capture the association between social media use and a broad range of both adolescent mental health and diet outcomes. This includes documenting their key recommendations for tackling this issue, and, where no recommendations exist, how their current adolescent health priorities might be leveraged to include the potentially negative impacts of social media use. International organisations with a focus on health, such as the World Health Organization and various bodies of the United Nations, are often seen as important sources of information that help to shape the policies of their member states. By focusing on such organisations, we believe that insights can be derived into the level of attention being paid to this global issue.

## Methods

### Study design

This study centred on a document analysis executed in two phases, combining summative content analysis with thematic analysis. One of three key approaches to qualitative content analysis laid out in a seminal paper by Hsieh and Shannon [9], summative content analysis begins by conducting searches within a given document for the occurrence of a set of pre-identified words or terms. Word frequency counts for each word or term are then calculated, while also identifying the context within which the word or term is used. To build on summative content analysis, thematic analysis can then be employed using the collected summative data to interpret its overall content and meaning through an interactive process between researchers [10]. The two-step methodological approach that forms the basis of the present study was inspired by a recent document analysis of health policies in the United Kingdom by Sleeman et al. [11].

In our study, we chose to pair summative content analysis with thematic analysis based on their combined ability to meet the overall aims of our work, i.e., to understand the extent to which the potentially negative mental health and diet effects of social media use among adolescents are being acknowledged by leading international child and adolescent health organisations in their recent strategies, action plans, policies and guidelines related to adolescent social media use, mental health and/or diet; to identify these organisations’ key recommendations for tackling this issue; and, where no such recommendations exist, to identify how existing priorities for child and adolescent health can be leveraged to include recognition of the potentially negative impacts of social media use among young people [9].

### Document inclusion criteria

Although not a systematic review, at the outset of our study we developed a set of inclusion criteria for the documents we would evaluate, in order to ensure a degree of comparability across our sample. As we aimed to gain a broad understanding of how international organisations are acknowledging and/or acting upon the growing evidence on the relationship between social media and various adolescent mental health and diet outcomes, we chose to include documents that were exclusively focused on adolescent social media use, adolescent mental health, and adolescent diet, as well as those that looked at any combination of these three topics. If documents focused on the general population but had dedicated sections related to adolescents, these were also considered. For the purposes of feasibility and given the rapidly evolving nature of social media, we chose to focus only on documents that had been published in the last five years. To this end, documents were considered for inclusion if they met the following criteria: i) strategies, action plans, policies or guidelines put forth by international bodies to address adolescent mental health, adolescent diet, adolescent social media use, or some combination of any of these three areas (as evidenced by their titles and abstracts/executive summaries); and ii) published between January 2018 and July 2023.

### Data collection

Data collection occurred between June and July 2023 and was carried out by two independent researchers (KCM and CK). To begin, web searches for relevant documents were conducted on a targeted set of international organisations’ websites (i.e., United Nations, UNICEF, UNESCO, European Commission, World Health Organization and its six regional offices, African Union, Gulf Cooperation Council, and the Organization for Economic Co-operation and Development Library), all chosen for their relevance to topics including child and/or adolescent health and young people’s engagement with the digital environment. To reduce the likelihood of missing documents issued by other relevant international organisations, this process was supplemented by searches on Google. For both website-specific and Google searches, a set of simplified keywords were amended from the search strategy utilised in the aforementioned systematic review by Blanchard et al. [7] to capture our three core concepts of interest, namely social media, adolescent mental health and adolescent diet, while also adding keywords for the type of document we were interested in. Ultimately, these keywords included “adolescents” OR “children” OR “youth” AND “social media” OR “digital environment” AND “mental health” AND “diet” AND “strategy” OR “action plan” OR “policy” OR “guideline”. The list of documents initially obtained through this search process was then discussed by the two researchers to determine a final list of included documents.

Once our set of included documents was finalised, PDF copies of each document were loaded into EPPI Reviewer 6 [12], a web-based literature review software. A data extraction sheet was programmed into EPPI Reviewer 6 [12] that allowed for extracting basic information on each document (i.e., the organisation(s) that had published the document; the publication year; the title of the document; the stated purpose or aim of the document), as well as information required for both summative content and thematic analyses (i.e., the specific word(s)/term(s) identified related to social media, adolescent diet and adolescent mental health; the number of times each word was used; the page number and section(s) where the word(s) appeared; and the surrounding text (usually a paragraph)). All data collection was conducted using the document search function built into EPPI Reviewer 6 [12]. The words and terms pre-identified for these document searches were once again based on a modified list of search terms used as part of the systematic review by Blanchard et al. and can be seen in Table 1 [7]. To avoid overestimation, searches were limited to the main body or appendix text of each document, and excluded titles, footnotes and references. The rationale for including appendix material rested on the fact that many of the documents utilised these sections to expand on the details of the strategies and action plans outlined in the main body text of the document. Data extraction was conducted mainly by one researcher (KCM), with cross-checking of a sub-sample carried out by a second researcher (CK).

**Table 1:** Search terms for summative content analysis

Concept	Social Media	Diet	Mental Health
**Keyword**	social media	diet*	body image
	social network*	eat*	depressi*
	digital media	over-eat*	anxiety
	internet use	overeat*	anxi*
	cyberbull*	binge-eat*	self-esteem
		bing*	self esteem
		disordered-eat*	stress
		eating disorder*	lonel*
		unhealth*	interpersonal
		junk food*	
		fast food*	

Note: *At the end of words in this table symbolises truncation, which allows for searches to include all variations of that word's ending

To prepare for thematic analysis, the surrounding text of each pre-identified word/term found in a given document was reviewed to capture relevant policy recommendations and actions related to adolescent social media use, mental health and/or diet. These policy recommendations and/or actions were then entered into a Microsoft Excel V.16.89 spreadsheet [13].

### Data analysis

In line with the first phase of our methodological approach, data collected in the summative content analysis extraction form for each document on EPPI Reviewer 6 [12] were reviewed to calculate the frequency with which each of our pre-identified words/terms representing adolescent social media use, mental health and diet were used (either individually, in pairs, or with all three terms linked together), as well as the specific location within the document they appeared. In line with the second phase of our approach, i.e., thematic analysis, policy recommendations and/or actions entered into the Microsoft Excel V.16.89 spreadsheet [13] were first inductively coded based on the overarching aim or focus of the individual policy recommendation/action. As this resulted in a very large and diverse set of codes, following discussions between researchers (KCM and CK), this initial set of codes was refined into a smaller set that captured the overarching focus of the policy recommendations/actions in a more general way (for example: school-based mental health intervention, efforts to improve online content, etc.) that could be applied across the entire sample. In order to ensure the reliability of this smaller set of codes in representing the overall meaning of the full sample of policy recommendations/actions, inter-coder reliability (ICR) testing was carried out on random 25% samples of individual policy recommendations/actions by two coders (KCM and AA) with codes being refined until a Cohen’s Kappa of 0.84 (95% CI: 0.69–0.99) was attained [14]. The final set of agreed codes from across the entire sample were then organised into a set of overarching themes representing the emerging priorities for adolescent health evident across all documents, with a view to the original, full set of individual policy recommendations and/or actions captured. Themes were discussed and refined by two researchers (KCM and CK) until agreement was reached.

## Results

### Data sample

A total of 31 documents were included in the final analysis, covering the strategies, action plans, guidelines, and/or policies of a range of international organisations, including UNICEF (*n* = 9), the World Health Organization (*n* = 8), the United Nations (*n* = 3), the Pan American Health Organization (*n* = 2); the Organization for Economic Co-operation and Development (*n* = 2), the European Commission (*n* = 2), the European Parliament and Council of the European Union (EU) (*n* = 1), the Council of Europe (*n* = 1), UNESCO (*n* = 1), and joint publications by UNICEF and the World Health Organization as well as by the Gulf Cooperation Council and United Nations Population Fund, respectively (*n* = 1 each). The focus of these included documents ranged from adolescent mental health (*n* = 10), adolescent diet (*n* = 7), young people’s engagement with the digital environment (*n* = 7), adolescent health and wellbeing more broadly (*n* = 3), online food marketing to young people (*n* = 2), mental health in the digital environment (*n* = 1) and youth engagement (*n* = 1).

### Summative content analysis

**Fig. 1:**
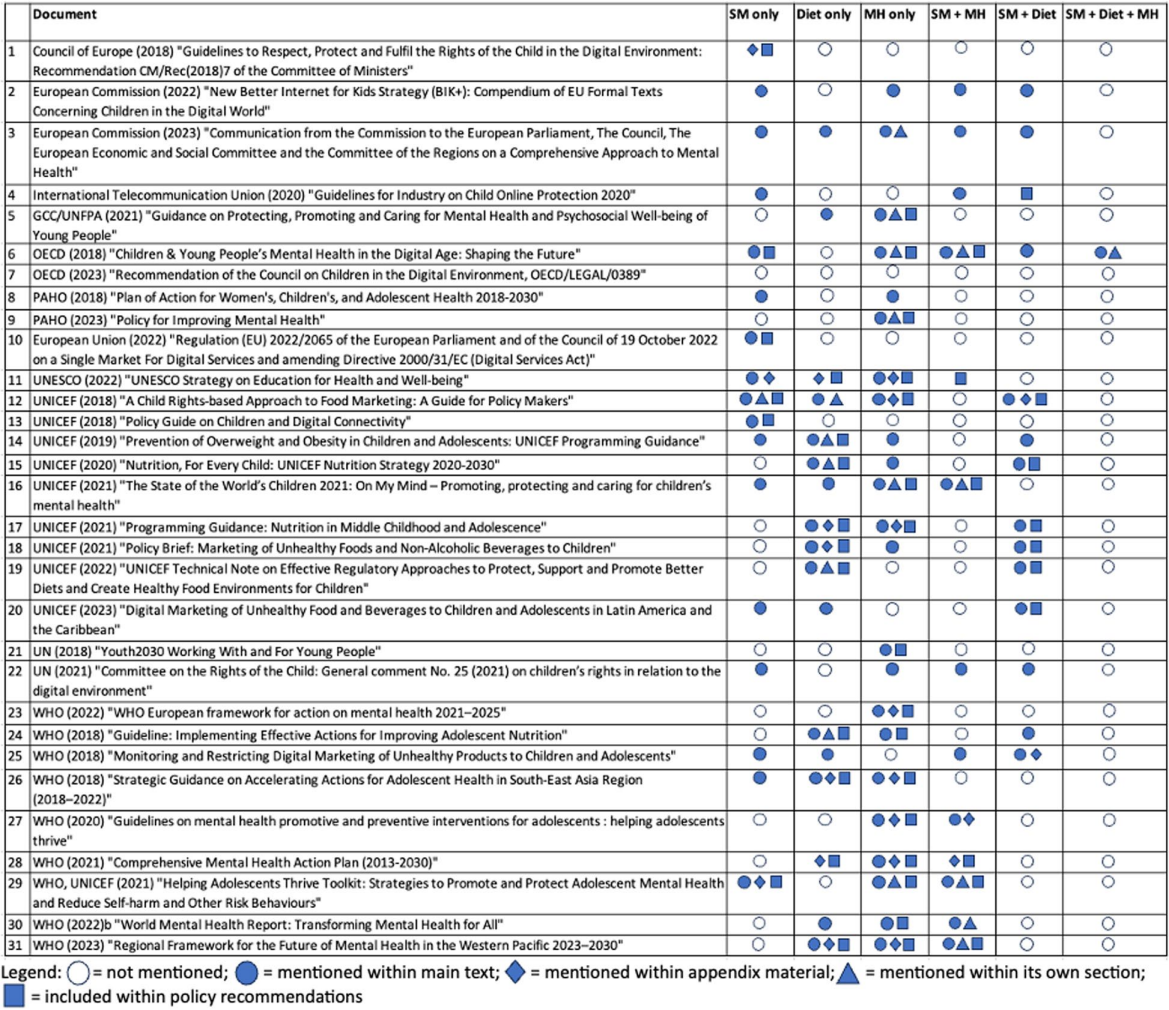
Policy content map based on summative content analysis. Note: SM = social media; MH = mental health. Sources: [15–45]

Figure 1 outlines the findings of the summative content analysis. Based on word frequency counts, mental health was the most common topic, with mentions in the main text/appendices or in dedicated sections within 23 of the 31 included documents. Individual mentions of diet or social media were the second most common topics, respectively, found in 17 documents each. The role of social media on diet was mentioned in 14 documents, followed by the role of social media on mental health, mentioned in 13 documents. Lastly, the role of social media on *both* mental health and diet was mentioned in the main text of only one document, though in that instance it was given a brief dedicated section in the introduction. Document-specific breakdowns of word/term frequency counts from the summative content analysis can be found in Supplementary Table 1.

When it came to the provision of policy recommendations or actions within the documents, these centred first and foremost on the domain of adolescent mental health (*n* = 16 documents), followed by diet (*n* = 10), social media and diet (*n* = 7), social media (*n* = 6), and social media and mental health (*n* = 6). No documents outlined recommendations or actions to address the impact of social media on *both* adolescent mental health and diet.

A total of 152 policy recommendations and/or actions were extracted from across the 31 included documents as part of the summative content analysis. Table 2 provides an overview of the overarching focus of policies that emerged across the included documents, presented by domain. Related to social media, these included regulations to protect children’s right to safety online, developing age-appropriate online content and materials to promote protecting oneself online, and limiting access to harmful online content and removing illegal content [15, 20, 27]. Other policy recommendations and/or actions related to social media included improving the ability of schools to tackle online abuse by equipping them with resources, training and support to roll out both preventative and protective measures [15], and developing parent training programmes as a means of home-based intervention [43].

**Table 2:** Overarching policy focus by domain

SM only	Diet only	MH only	SM + MH	SM + Diet	SM + Diet + MH
• Regulations and resources to protect children’s online safety • Enabling school- and home-based digital safety training	• School-based diet/nutrition interventions and knowledge promotion • Government-led diet/nutrition interventions focused on improving food environments	• Improved mental health governance for adolescents • School-led mental health initiatives • Engaging parents/caregivers in mental health support for adolescents • Targeted mental health care for adolescents	• School based interventions that combine mental health promotion with digital safety training • Monitoring/removing online content harmful to mental health • Expanding mental health care delivery platforms	• Restricting online advertising of unhealthy food and beverages • Social media-based nutrition interventions	-

*SM* social media, *MH* mental health

Recommendations related to diet were numerous and far-reaching, but centred heavily on school-based interventions, including an increased focus on diet and nutrition education in school curriculums, school-based support for disordered eating, and enhancing the food environment through the provision of healthy food options in schools [25, 28, 29, 31, 33, 38, 42, 45]. Government-led initiatives related to improving young people’s dietary outcomes were also frequently promoted, including enhancing food labelling requirements and product reformulation [33]; the creation of national guidance for school meals [28]; and regulating the sale and/or advertisement of unhealthy foods within and directly outside the school environment [28, 29, 33].

Recommendations related to mental health were also numerous and far-reaching, but generally included improved governance of mental health services through the allocation of sufficient budgets and resources to expand care and research targeting adolescents [19, 20, 30, 37, 42]; expanding school-based mental health support [22, 25, 37]; engaging parents and caregivers in promoting adolescent mental health [30, 43, 44]; and a specific focus on youth engagement in mental health initiatives [35, 37, 40, 41, 43].

Recommendations related to social media and mental health included school-based interventions such as digital awareness training focused on cyberbullying and social media addiction, as well as tools for navigating online disinformation [20, 25]; harnessing technological tools to monitor and/or remove online content that is harmful to adolescent mental health [30, 43]; and expanding mental health delivery platforms, including using social media as a tool in promotion and prevention strategies and offering digital mental health services as a means of overcoming stigma around accessing care [43, 45].

Finally, related to social media and diet, recommendations included restricting the online advertising of unhealthy food and beverages targeted at children and adolescents [18, 26, 29, 31–34]; improving the promotion of healthy diets and nutrition through social media campaigns [29, 31]; and engaging influencers and celebrities with a large child and adolescent following to promote healthy diets [28].

### Thematic analysis of emerging priorities for adolescent health

As detailed above, the 152 policy recommendations and/or actions extracted during summative content analysis were far ranging and often covered multiple aspects of adolescent social media use, mental health and/or diet. As such, thematic analysis was rooted in an iterative process whereby the specific aim(s) of each individual policy recommendation and/or action were amalgamated into a smaller set of codes that could capture recurring areas of focus across the entire sample. After discussions between reviewers (KCM and CK), this ultimately resulted in seven codes, including: community-based health interventions; government-led health interventions; school-based health interventions; home-based health interventions; focus on improving adolescent health promotion/prevention delivery; focus on online content (both marketing and non-marketing); and applying a youth focus to health interventions. Based on this coding process, four inter-related themes emerged that broadly represent the key priorities for improving adolescent health evident across all of the documents and their numerous policy recommendations and/or actions (see Table 3 for coding frame). Although not specific to social media’s impact on both adolescent health and diet, each of these themes signal important leverage points with which future policy recommendations and actions in this area can align. These themes include health services provision, knowledge promotion, content regulation, and environmental improvements.

**Table 3:** Coding frame emerging from thematic analysis

Themes	Health Services Provision	Knowledge Promotion	Content Regulation	Environmental Improvements
**Codes**	Applying a youth focus to health interventions	Applying a youth focus to health interventions	Applying a youth focus to health interventions	Applying a youth focus to health interventions
	Government-led health interventions	Community-based health interventions	Focus on online content (both marketing and non-marketing)	Focus on online content (both marketing and non-marketing)
	Home-based health interventions	Focus on online content (both marketing and non-marketing)	Focus on improving adolescent health promotion/prevention delivery	Government-led health interventions
	Focus on improving adolescent health promotion/prevention delivery	Government-led health interventions		Focus on improving adolescent health promotion/prevention delivery
	School-based health interventions	Home-based health interventions		School-based health interventions
		Focus on improving adolescent health promotion/prevention delivery		
		School-based health interventions		

Table 4 provides a description of each of the four themes identified and presents representative quotes of policy recommendations and/or actions captured under each theme. Items under the health services provision theme focused on expanding and/or strengthening health interventions and services so they are better able to reach adolescents and more tailored to the needs of this age group. This included identifying new delivery methods for mental health and diet interventions within settings meaningful to adolescents such as school, community spaces or online, and having the government take a more active role in ensuring all adolescents have access to the care they need, including by prioritising universal coverage for adolescent mental health care. Items under the knowledge promotion theme focused on increasing knowledge, awareness and informational resources needed to improve and protect adolescent health, both online and offline. This included incorporating a focus on healthy eating in school curriculums; using social behaviour change communication to improve adolescents’ diet and increase their physical activity; and creating nutrient intake guidelines specifically for the adolescent age range. Items under the online content regulation theme largely aligned with a rights-based approach, with a specific focus on protecting young peoples’ rights to health and personal safety online. This included eliminating or restricting children and adolescents’ access to certain types of content, including content that promotes self-harm or advertises unhealthy food and beverages. Lastly, items under the environmental improvement theme focused on making changes to adolescent spaces, both online and offline, in order to optimise health outcomes. This included imposing regulations on the types of food that can be sold in and around schools; working directly with social media influencers and celebrities to promote empowerment and change online; and reshaping community spaces to promote mental health.

**Table 4:** Priority areas for improving adolescent health

Theme	Description	Examples of Policy Recommendations/Actions
Health Services Provision	Items under this theme focus on expanding or strengthening health interventions and services so they are better able to reach adolescents and are appropriate to the needs of this age group	Using digital media to deliver mental health services: • *WHO/UNICEF Helping Adolescents Thrive Toolkit*: "Implementation considerations for adolescents with emotional problems (existing psychological symptoms but no existing diagnosis: Digital, face-to-face and combined approaches are feasible and effective options for delivery [of mental health services]. […] Delivering interventions using digital media could mitigate stigma."[43] Universal coverage for mental health care: • *Youth2030 Working With and For Young People*: "Priority Areas of the UN Youth Strategy: Support youth-friendly mental health services: Ensure accessibility of youth-friendly mental health services, within the greater context of universal health coverage." [35] • *WHO European framework for action on mental health 2021–2025*: Responding to mental health challenges in the WHO European Region: strategic objectives and actions: “[M]oving towards universal health coverage.” [37]
Knowledge Promotion	Items under this theme focus on delivering information about safe online engagement, mental health promotion/prevention strategies and ways to improve diet/nutrition outcomes for young people	Promoting nutrition and physical activity in school • *Nutrition, For Every Child: UNICEF Nutrition Strategy 2020–2030*: “Nutrition education in school curricula: UNICEF advocates for and supports policies, strategies and programmes that enhance school curricula to improve knowledge and skills about good nutrition and physical activity among school-age children and adolescents. This involves promoting nutrition education and physical education in primary and secondary school curricula and improving the capacities of teachers and school managers to deliver nutrition education and promote good nutrition and physical activity.”[29] Social behaviour change communication on healthy diets and physical activity • *Prevention of Overweight and Obesity in Children and Adolescents: UNICEF Programming Guidance*: “Social mobilization and social and behaviour change. Communication for families and adolescents on healthy diet and physical activity through a range of channels such as social networks, peer groups and social media.” [28]
Online Content Regulation	Items under this theme focus on protecting children’s rights to health and safety by restricting or removing online content that could be seen as harmful, including advertising	Removing illegal content: • *Council of Europe Guidelines to Respect, Protect and Fulfil the Rights of the Child in the Digital Environment: Recommendation CM/Rec(2018)7 of the Committee of Ministers*: "115. States should engage business enterprises, such as service providers and social network providers, to play an active role in preventing and deleting illegal content, as determined by law or by a judicial or other competent authority." [15] Restricting marketing of unhealthy food and beverages to children • *UNICEF Policy Brief: Marketing of Unhealthy Foods and Non-Alcoholic Beverages to Children*: “Children are increasingly frequent and social media users and so it makes sense that unhealthy food and beverage corporations are tapping into these new promotional avenues. To properly address the harmful influence of unhealthy food and beverage marketing these multiple marketing channels need to be addressed in restrictions.” [32]
Environmental Improvement	Items under this theme focus on improving young people’s environments, including at home, school and online, in order to improve health outcomes. Several items under this theme focus on government-led initiatives as well as incorporating a youth perspective	Regulations for the school food environment • *UNICEF Technical Note on Effective Regulatory Approaches to Protect, Support and Promote Better Diets and Create Healthy Food Environments for Children*: “Food in schools: Mandatory policies and standards around food and beverages provided or available in [and] around daycare centres, preschools and schools.” [33] Reshaping public spaces to promote mental health • *WHO World Mental Health Report: Transforming Mental Health for All*: “Paths to Transform: Reshape physical, social and economic characteristics of different environments for mental health, including: homes, schools, workplaces, health care services, communities, and natural environments […].” [44]

## Discussion

Adolescence is a critical period, shaped by the events of prenatal and early childhood development and the onset of puberty, with important knock-on effects for later life health outcomes [46]. In the face of an increasingly digital world, and with social media use occurring at increasingly younger ages, it is important to understand the role that social media has in shaping adolescent health outcomes. As such, this study aimed to build on recent work related to the potentially harmful impact of social media use on adolescent mental health and diet outcomes by exploring how these relationships are being captured in the recent strategies, action plans, policies and guidelines put forth by international organisations with significant influence in the areas of child and adolescent health. Overall, our analysis of 31 relevant documents from across 11 organisations showed that only one has drawn attention to the link between social media use and both adolescent mental health and diet outcomes, while none currently provides policy recommendations or action plans to tackle this emerging public health issue. By reviewing the wider range of policy recommendations and proposed actions for improving adolescent health included across these 31 documents, however, four inter-related priority areas were identified that may be leveraged in future policies aimed at addressing the role of social media use on adolescent mental health and diet. These include: 1) improving and expanding the provision of health services for young people; 2) strengthening knowledge promotion efforts to increase young people’s uptake of health interventions; 3) regulating online content so that age-inappropriate content cannot reach children; and 4) making critical improvements to young people’s different lived environments in order to optimise their mental and physical health.

Existing approaches currently being developed by international organisations were identified within this study that both exemplify these inter-related priority areas for adolescent health and can also act as a starting point for increasing action on the potentially harmful impacts of adolescent social media use on both mental health and diet. For example, the CLICK framework is an innovative tool outlined in an included 2018 report from WHO’s Regional Office for Europe entitled “Monitoring and Restricting Digital Marketing of Unhealthy Products to Children and Adolescents” which aims to increase knowledge promotion, regulate online content and generally improve young people’s online environments [39]. Currently designed as a way to monitor children’s exposure to the digital marketing of unhealthy products (including alcohol, tobacco, electronic cigarettes and foods high in saturated fat, salt and sugar), CLICK is an acronym that stands for Comprehend the digital ecosystem; Landscape of campaigns, Investigate exposure; Capture on-screen; and Knowledge sharing. At the root of the CLICK framework are three proposed policy pre-requisites that countries should implement, including: 1) age verification of online users; 2) tagging marketing campaigns that should not be viewed by children; and 3) regulation to ensure collected data is used to prevent children’s access to age-restricted advertising. Though developed to prevent the harmful dietary and/or wider health outcomes of advertising, this kind of tool could be readily expanded to also target social media content with potentially harmful consequences for adolescent mental health.

Development and expansion of a tool like the CLICK framework is especially pertinent given the recent ramp up of influencer-based marketing on social media, which reached US$24 billion in 2024, a nearly four-fold increase since 2019 [47, 48]. As previously mentioned, youth participants in systems mapping exercises as part of the CO-CREATE project identified the negative impact of social media influencers on their mental health as well as their subsequent diet and exercise habits, and it is clear from recent data that young people’s knowledge related to influencer marketing requires strengthening [5, 6]. Specifically, in 2022, the UK’s communications regulator Ofcom found that while 70% of 12–17-year-olds were able to correctly identify that an influencer was promoting a product due to a paid partnership, only 42% said that this was the only reason for the promotion, with the rest indicating some other reason, such as the influencer having a genuine interest in the product [49].

While countries such as the United States and the United Kingdom have regulations in place to ensure that social media influencers are transparent in their advertising practices by clearly identifying when their posts are paid promotions, few countries have enacted legislation that targets the specific content being produced or promoted by influencers. Notable exceptions include a 2021 law passed in Norway that requires influencers to label retouched images, and a 2023 law in France that requires influencers to disclose the use of image retouching software or filters, while also prohibiting content that promotes cosmetic surgery, refraining from medical treatment, and products containing nicotine or gambling, as well as a ban on content featuring animals it is illegal to own [50, 51]. At the same time, leading social media companies such as Meta (who own popular applications such as Facebook and Instagram) and X (formerly Twitter) have recently taken steps to remove the independent fact-checking of content on their platforms, instead leaving indications of the accuracy of information being shared up to individual user comments [52].

More broadly, only Australia, the European Union, the United Kingdom and Singapore currently have national/multi-national-level legislation in place related to digital safety with a focus on protecting children from harmful online content. In Australia, both the Enhancing Online Safety Act 2015 and the expanded Online Safety Act 2021 focus on holding online service providers accountable for the safety of their users, including by developing codes to regulate content that is inappropriate for children (similar to the approach promoted by the CLICK framework) [53]. In 2025, Australia also became the first country in the world to ban social media use for children under 16 years of age, with France moving towards a similar ban for children under 15 years of age in 2026 and several other countries currently considering similar legislation at the time of this writing [54, 55]. In the EU, the 2022 Digital Services Act (DSA)—documentation of which was included among the 31 documents in this study—aims to foster a safer digital environment where the fundamental rights of users are protected, while also establishing a more equitable landscape within which businesses can operate [56]. The DSA also explicitly aims to target users’ exposure to illegal content and disinformation, as well as protecting children online [57]. Similarly, in the UK, the 2023 Online Safety Act is a regulatory framework that imposes requirements on providers of online services to intervene and minimise risks of harm due to illegal content and activity as well as content and activity which is harmful to children. It is intended to ensure that online services are regulated such that they are safe by design and operated so that, among other things, children are afforded greater protection [58]. Finally, Singapore’s 2023 Online Safety Act has been promoted as a regulatory effort to ensure a safe online environment for all users, while also having a specific focus on protecting children. While all of these examples indicate a potentially positive step towards improving the mental health and diet outcomes resulting from adolescents’ social media use, further research is required to assess their implementation and impact in these specific areas.

While a key strength of the two-step methodological approach used in this study was the ability to include a wide range of international organisations’ recent publications and an even greater number of topic areas relevant to adolescent social media use, mental health and diet, it is important to also consider the limitations of this research. Firstly, interpretation of results should be tempered by the fact that the documents included in our sample were not like for like, but rather focused on a number of different aspects of social media use, mental health and diet, all with different aims and objectives. As such, the key takeaway from this research should centre on the fact that there is currently limited attention from leading international organisations on social media’s potentially harmful impacts on both adolescent mental health and diet outcomes, but that there exist key areas of alignment between this emerging issue and the current child and adolescent health priorities of these organisations. Another limitation of this research is that by focusing only on international organisations, our findings may exclude important policy actions being taken at national, sub-national or local levels. Similarly, in limiting our search to documents published within the last five years in an effort to keep pace with the rapidly changing nature of social media and the digital environment, it is possible that previous international efforts to combat negative mental health and diets effects of social media use might have been overlooked, although we made an effort to highlight other relevant initiatives within our discussion.

In terms of implications for future policy, the findings of this study indicate the need for international organisations focused on child and adolescent health—and the member states that look to them as thought leaders—to begin to engage in active communication, if not targeted action, on the potential role of social media use in negative adolescent mental health and diet outcomes. This could be done by drawing on and promoting existing research; funding new research specifically aimed at establishing casual links between adolescents’ social media use and poor health outcomes; and engaging directly with young people in order to understand how to best protect their health and wellbeing. These efforts can all be made while directly linking to ongoing priority areas around improving/expanding health services for young people, enhancing their knowledge around how to maintain and improve their health, regulating the content they see online, and improving both their online and physical environments.

## Conclusions

The findings of this study indicate that emerging evidence related to the harmful effects of social media use on both adolescent mental health and diet is largely missing among the outputs of prominent international organisations focused on child and adolescent health. However, our findings also show that these organisations are actively engaged in the important work of bettering the health of young people via policy recommendations in the areas of improved health service provision, knowledge promotion, online content regulation, as well as through changes to the environments in which young people live, study, work and socialise. These are important leverage points that can be used to incorporate interventions that target some of the more complex outcomes of young lives being lived increasingly online. While future research is needed to establish a causal link between social media exposure and harmful mental health and diet outcomes among adolescents, calls to action from leading actors in public health and recent steps being taken across Europe as well as in Australia and Singapore indicate that this is an emerging public health issue that cannot be ignored, and that attention is needed from the international organisations we look to for global leadership in matters of health and wellbeing.

## Supplementary Information

Supplementary Material 1.

## Data Availability

The datasets used and/or analysed during the current study are available from the corresponding author on reasonable request.

## Abbreviations

CO-CREATE: Confronting obesity: Co-creating policy with youth
UK: United Kingdom
UNICEF: United Nations Children's Fund
UNESCO: United Nations Educational, Scientific and Cultural Organization
ICR: Inter-coder reliability
EU: European Union
SM: Social Media
MH: Mental Health
CLICK: Comprehend the digital ecosystem, Landscape of campaigns, Investigate exposure, Capture on-screen, and Knowledge sharing
DSA: Digital Services Act
